# Phenotypic screening for metallo-β-lactamases and extended-spectrum β-lactamases in *Pseudomonas aeruginosa* using the VITEK-2 AST-N443 and Phoenix NMIC-486 cards

**DOI:** 10.1128/spectrum.02235-26

**Published:** 2026-09-23

**Authors:** Stefano Mancini, Linda Mueller, Silvio D. Brugger, Helena M. B. Seth-Smith, Peter M. Keller, Natalia Kolesnik-Goldmann, Muhammad Ali Syed, Tim Roloff, Oliver Nolte

**Affiliations:** 1 Institute of Medical Microbiology, University of Zurich 27217, Zürich, Switzerland; 2 Istituto di Medicina di Laboratorio EOC, Bellinzona, Switzerland; 3 Department of Infectious Diseases and Hospital Epidemiology, University of Zurich, University Hospital Zurich 27217, Zürich, Switzerland; 4 Department of Laboratory Medicine, Clinical Bacteriology/Mycology, University of Basel 27209, Basel, Switzerland; 5 Department of Microbiology, The University of Haripur 384987, Haripur, Pakistan; JMI Laboratories, North Liberty, Iowa, USA

**Keywords:** carbapenemase detection, CRPA, VITEK, Phoenix, BL/BLI combinations

## Abstract

*Pseudomonas aeruginosa* can develop carbapenem resistance through chromosomal mechanisms and/or by acquiring plasmid-mediated carbapenemases, including the most prevalent metallo-β-lactamases (MBLs) and/or extended-spectrum β-lactamases (ESBLs). Conventional β-lactam susceptibility testing does not reliably discriminate MBL/ESBL-producing carbapenem-resistant *P. aeruginosa* (MBL/ESBL-CRPA) from non-carbapenemase-producing isolates, here referred to as AmpC-hyperproducers. However, these groups exhibit distinct susceptibility patterns to novel β-lactam/β-lactamase inhibitor (BL/BLI) combinations, including ceftolozane/tazobactam (C/T), ceftazidime/avibactam (C/A), imipenem/relebactam (I/R), and meropenem/vaborbactam (M/V). We investigated potential screening cut-offs for detecting MBL/ESBL production in CRPA using the VITEK-2 AST-N443 and Phoenix NMIC-486 cards. Antimicrobial susceptibility testing was performed using the VITEK-2 AST-N443 and Phoenix NMIC-486 cards on 135 whole-genome-sequenced international CRPA isolates resistant by disc diffusion to ceftazidime, cefepime, imipenem, and/or meropenem. The collection included 71 MBL-producing isolates, of which nine co-produced ESBLs, as well as one KPC-2-, one GES-5-, and twelve ESBL-only producers. The remaining 50 represented AmpC-hyperproducers. MIC distributions were analyzed to identify discriminatory screening cut-offs. C/T was the most discriminatory BL/BLI combination for differentiating MBL/ESBL-CRPA from AmpC-hyperproducers. For the VITEK-2 AST-N443, a C/T MIC ≥32 mg/L achieved 100% sensitivity for MBL±ESBL detection (71/71) and 91.7% sensitivity for ESBL-only producers (11/12), with 82% specificity (41/50). For the Phoenix NMIC-486, a C/T MIC >8 mg/L achieved 100% sensitivity for both MBL and ESBL detection, with 80% specificity (40/50). Combining additional BL/BLI agents did not further improve specificity. Elevated C/T MICs represent reliable phenotypic screening markers for MBL and ESBL production in CRPA using both the VITEK-2 AST-N443 and Phoenix NMIC-486 systems.

**IMPORTANCE:**

Rapid identification of the underlying resistance mechanism in carbapenem-resistant *Pseudomonas aeruginosa* (CRPA) is essential for effective treatment and infection control. Differentiating high-level resistance acquired via plasmid-borne carbapenemases and/or extended-spectrum β-lactamases (ESBLs) from resistance driven by intrinsic mechanisms, including AmpC-type β-lactamase hyperproduction, remains challenging because conventional antimicrobial susceptibility testing (AST) using classic β-lactams cannot reliably separate these phenotypes. This distinction is relevant because many recently introduced β-lactam/β-lactamase inhibitor combinations are inactive against metallo-β-lactamase (MBL) carbapenemase-producing isolates but may remain active against strains in which resistance is driven by intrinsic mechanisms. Detection of acquired carbapenemases may also trigger enhanced infection-control measures and epidemiological investigations. Our study identifies reliable screening cut-offs for MBL- and ESBL-production in CRPA using standard VITEK 2 and Phoenix automated AST panels. This readily available approach may accelerate detection, support routine laboratory workflows, reduce unnecessary confirmatory testing, and facilitate earlier targeted therapy and infection-control interventions in laboratories using these systems worldwide without requiring additional specialized testing or infrastructure.

## OBSERVATION

Carbapenem-resistant *Pseudomonas aeruginosa* (CRPA) is classified by the World Health Organization as a critical-priority pathogen because of its role in healthcare-associated infections and limited therapeutic options (1). Resistance to carbapenems and other antipseudomonal β-lactams can arise through mechanisms with therapeutic and infection-control implications (2). Broad β-lactam resistance may result from activation of chromosomal mechanisms, including AmpC derepression combined with porin loss and/or efflux pump overexpression, and/or from acquisition of β-lactamases, such as metallo-β-lactamases (MBLs), class A carbapenemases (e.g., GES), or extended-spectrum β-lactamases (ESBLs), the latter necessarily occurring in combination with additional resistance mechanisms (3). This distinction is relevant because many recently introduced β-lactam/β-lactamase inhibitor (BL/BLI) combinations are inactive against MBL-producing isolates, whereas they may remain active against multidrug-resistant strains driven primarily by intrinsic mechanisms; thus, rapid differentiation enables the timely initiation of targeted therapy. Detection of an acquired carbapenemase may also trigger enhanced infection-control interventions and epidemiological investigations (4, 5). Conventional susceptibility testing with classic β-lactam agents cannot reliably differentiate acquired β-lactamases from chromosomal resistance mechanisms. In contrast, BL/BLI combinations, including ceftolozane/tazobactam (C/T), imipenem/relebactam (I/R), ceftazidime/avibactam (C/A), and meropenem/vaborbactam (M/V), exhibit distinct activity profiles that may provide diagnostically useful phenotypic signatures (6, 7). We investigated whether these novel BL/BLI agents incorporated into two widely used automated MIC-based antimicrobial susceptibility testing (AST) systems, the VITEK-2 AST-N443 and Phoenix NMIC-486 cards, could provide a simple phenotypic screening approach for the detection of acquired MBL and ESBL in CRPA. This study did not assess MIC accuracy against reference broth microdilution; findings are therefore specific to the VITEK 2 AST-N443 and Phoenix NMIC-486 cards evaluated.

### Isolate collection and automated AST

The collection comprised 135 non-duplicate CRPA isolates. Of these, 128 isolates were previously described by Mancini et al. and were selected to represent diverse resistance mechanisms (6). Additional CRPA strains were isolated at the Institute of Medical Microbiology, University of Zurich, between 2024 and 2025 (Table S1). Genome sequences generated at the IMM for isolates CRPAZU02-CRPAZU145 have been deposited in the ENA under project accession PRJEB82838. The collection encompassed 71 MBL-producers, of which two additionally carried a carbapenemase (OXA-181 and GES-5, respectively), and eight additionally harbored an ESBL. Further isolates included 12 isolates carrying class A ESBLs without carbapenemases, 1 KPC-2 producer, 1 GES-5 carbapenemase producer, and 50 isolates lacking acquired carbapenemase or ESBL genes, also referred to as “AmpC-hyperproducers.” Isolates originated from diverse clinical and geographical sources and were typically resistant to multiple antipseudomonal β-lactams, including imipenem and/or meropenem. All isolates underwent AST using the VITEK-2 AST-N443 and Phoenix NMIC-486 cards. MICs are provided in Table S1.

### Ceftolozane-tazobactam as the primary screening marker

**Fig 1:**
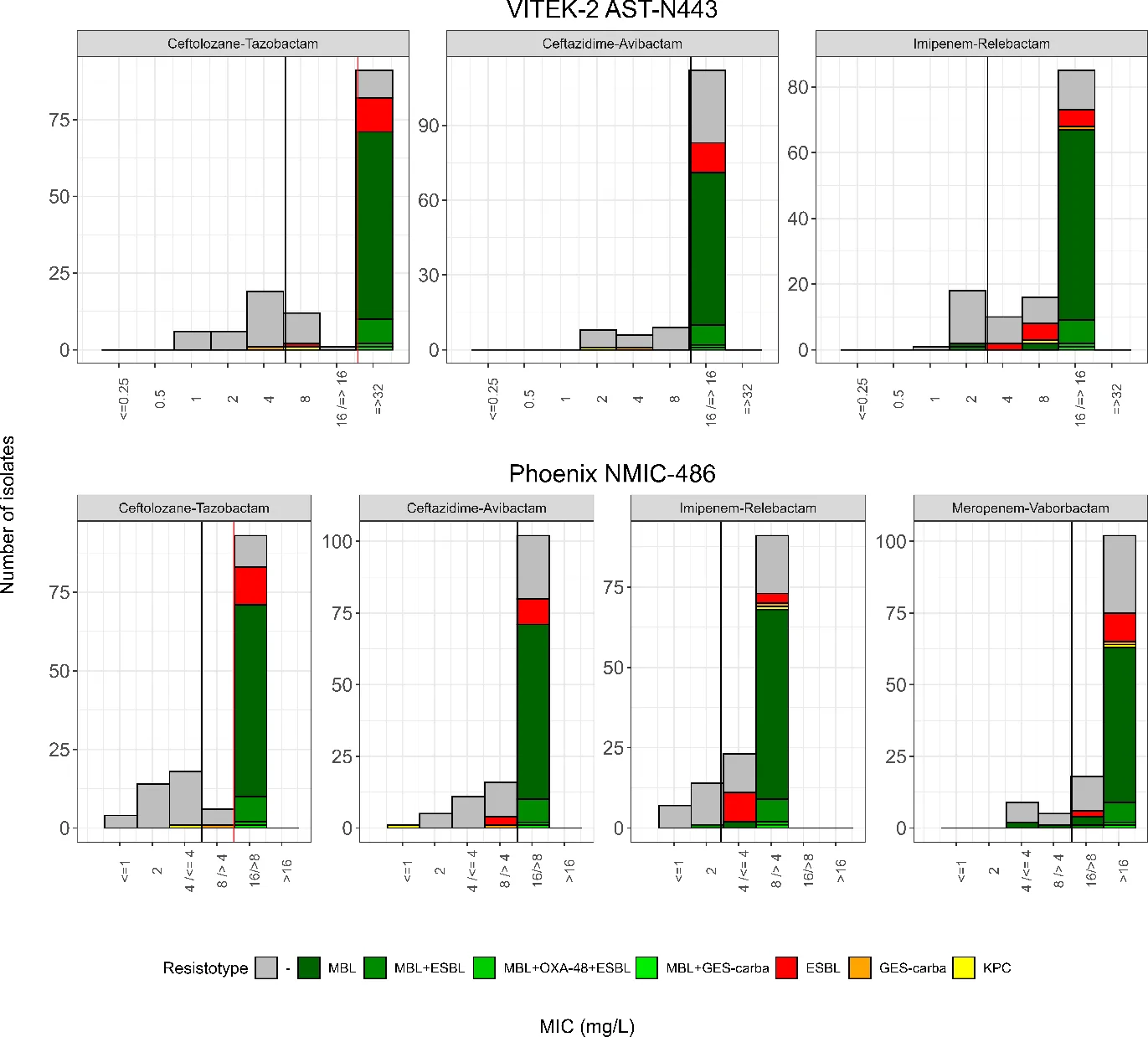
MIC distribution of novel BL/BLI combinations obtained with the VITEK-2 AST-N443 (top) and Phoenix NMIC-486 (bottom) cards. Vertical black lines indicate EUCAST clinical breakpoints; vertical red lines indicate the proposed card-specific screening thresholds for MBL/ESBL detection. Bars are stratified according to the underlying β-lactamase background (resistotype).

C/T was the most discriminatory BL/BLI combination for differentiating MBL- and/or ESBL-producing CRPA from AmpC-hyperproducers using both AST-systems (Fig. 1). For the VITEK-2 AST-N443 card, a C/T MIC ≥32 mg/L achieved 100% sensitivity for MBL±ESBL producers (71/71) and 91.7% sensitivity for ESBL-only producers (11/12), while maintaining 82% specificity (41/50) against AmpC-hyperproducers. Nine AmpC-hyperproducers also exhibited C/T MICs ≥32 mg/L, whereas the KPC-2-, the GES-5-, and one ESBL-producer displayed lower MICs. In contrast, I/R and C/A showed poorer discrimination between resistotypes. Although most MBL±ESBL producers exhibited I/R MICs ≥16 mg/L (67/71, 94.4%), some MBL-producers (4/71, 5.6%), and most ESBL-only producers (7/12, 58.3%) displayed lower MIC values that overlapped substantially with those observed among AmpC-hyperproducers. Similarly, all MBL±ESBL and ESBL-only producers exhibited C/A MICs ≥16 mg/L (71/71 and 12/12, respectively); however, a considerable proportion of AmpC-hyperproducers also displayed elevated C/A MICs (29/50, 58%), substantially compromising specificity. For the Phoenix NMIC-486 card, a C/T MIC >8 mg/L achieved 100% sensitivity for both MBL-producing (71/71) and ESBL-producing isolates (12/12), with a specificity of 80% (40/50). As observed with the VITEK-2 card, all MBL±ESBL-producers (71/71, 100%) exhibited the highest measurable C/A MIC (>8 mg/L). However, elevated C/A MICs were also common among AmpC-hyperproducers (22/50, 44%), while some ESBL-only producers displayed lower MIC values (3/12, 25%), limiting discriminatory performance. Similarly, although most MBL±ESBL-producers exhibited elevated I/R (68/71, 95.8%) and M/V (63/71, 88.7%) MICs, these MIC ranges overlapped considerably with those observed among AmpC-hyperproducers (18/50, 36% and 27/50, 54%, respectively). Conversely, a subset of MBL±ESBL and ESBL-only producers displayed lower I/R (3/71 MBL±ESBL, 4.2%, 9/12 ESBL-only, 75%) and M/V (8/71 MBL±ESBL, 11.3%, 2/12 ESBL-only producers, 16.7%) MICs, further reducing sensitivity. Overall, neither C/A, I/R, nor M/V provided the degree of phenotypic separation achieved by C/T on either automated platform.

### Limited added value of companion BL/BLI agents

**Fig 2:**
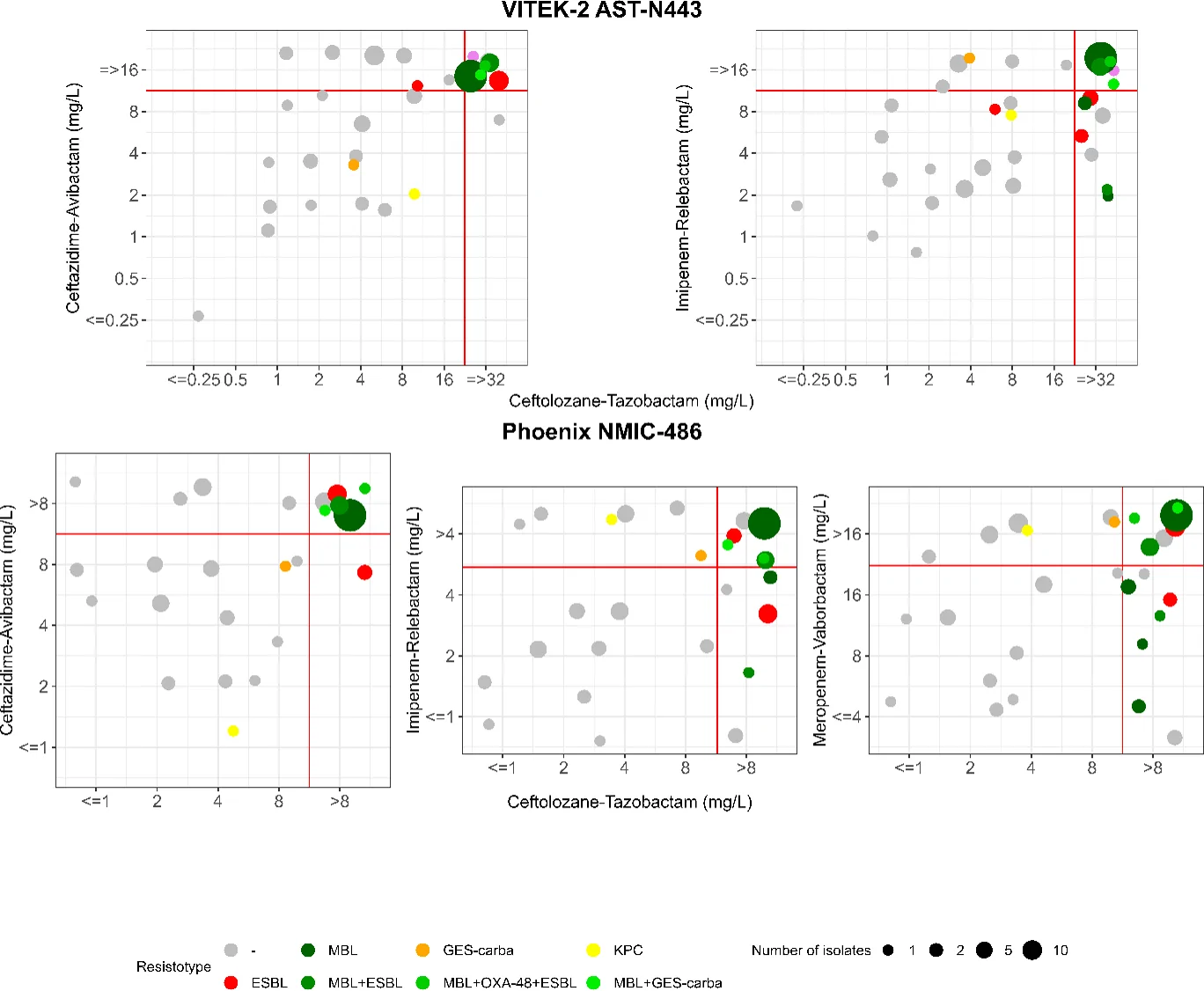
Bivariate MIC plots comparing ceftolozane-tazobactam with ceftazidime/avibactam and imipenem-relebactam on VITEK-2 AST-N443 (top), and with ceftazidime/avibactam, imipenem-relebactam, and meropenem/vaborbactam on Phoenix NMIC-486 (bottom). Red lines delineate the proposed upper-MIC screening zones associated with acquired carbapenemase and ESBL backgrounds. Point colors indicate the underlying β-lactamase background (resistotype), and point size corresponds to the number of isolates sharing the same MIC combination.

We then investigated whether information from additional BL/BLI agents among C/T-positive isolates could improve specificity and thereby reduce unnecessary confirmatory testing. On the VITEK-2 AST-N443 card, elevated C/A and I/R MICs generally clustered within the C/T-positive population (Fig. 2). Among isolates with C/T MICs ≥32 mg/L, all but one AmpC-hyperproducers also exhibited C/A MICs ≥16 mg/L, resulting in only a modest increase in specificity from 82% (41/50) to 84% (42/50) when both criteria were combined. In contrast, I/R MICs showed variability within the target population and therefore provided no additional discriminatory value. The Phoenix NMIC-486 yielded concordant results. Although elevated C/T MICs frequently co-occurred with high C/A, I/R, or M/V MICs, these companion BL/BLI agents exhibited some MIC overlap between carbapenemase-/ESBL-producing isolates and AmpC-hyperproducers. Consequently, none of the additional BL/BLI agents meaningfully improved specificity when applied as secondary screening criteria.

### Relation to the previous disc-diffusion analysis and proposed workflow

Consistent with previous analyses using automated susceptibility systems and disc diffusion (DD), both VITEK-2 AST-N443 and Phoenix NMIC-486 confirmed the strong screening performance of C/T for identifying CRPA enriched for acquired carbapenemase and/or ESBL backgrounds (6–9). However, in contrast to previous findings obtained by DD, MIC-based I/R readouts did not provide sufficient additional discriminatory power to confirm MBL production (4). This suggests that the first screening step of the previously proposed algorithm translates well to automated systems, whereas the downstream confirmatory steps remain method-dependent. Accordingly, CRPA isolates with elevated C/T MICs on VITEK-2 AST-N443 or Phoenix NMIC-486 should undergo confirmatory testing for MBL production. This may be performed using rapid immunochromatographic assays, such as Carba-5 or O.K.N.V.I. RESIST-5, or molecular methods, including PCR- or LAMP-based assays (10, 11). Alternatively, or following negative rapid confirmatory testing, laboratories may consider DD-based testing with C/T and I/R, as previously proposed, as a simple and low-cost phenotypic method to support the detection of (rare) MBL-producing isolates (6). Biochemical or carbapenem-inactivation assays, such as the Carba NP test, the modified carbapenem-inactivation method (mCIM), and the EDTA-mCIM, may also be used to confirm carbapenemase activity (12, 13).

### Biological basis of the C/T screening signal

Ceftolozane is highly stable against intrinsic AmpC, but it remains susceptible to hydrolysis by MBLs and several class A-ESBLs; tazobactam is likewise inactive against MBLs but may partially inhibit intrinsic AmpC. This explains the superior screening performance of C/T, although AmpC overexpression combined with structural alterations resulting in higher affinity to ceftolozane may also cause elevated MICs in isolates lacking acquired ESBL/carbapenemase genes (14–18).

### Conclusion

In conclusion, C/T MICs represent a robust and readily available phenotypic screening marker for MBL- and ESBL-producing CRPA isolates on both the VITEK-2 AST-N443 and Phoenix NMIC-486 systems. Although elevated C/T MICs require confirmatory testing, their integration as a reflex trigger within routine automated AST workflows may support earlier detection of clinically and epidemiologically relevant resistance mechanisms while enabling more targeted use of confirmatory assays. In regions with a high prevalence of class A carbapenemases, C/T should be further validated as a phenotypic screening marker in larger strain collections.

## SUPPLEMENTAL MATERIAL

Table S1 spectrum.02235-26-s0001.docx Genetic features and minimum inhibitory concentrations of the CRPA included in the study.

ASM does not own the copyrights to Supplemental Material that may be linked to, or accessed through, an article. The authors have granted ASM a non-exclusive, world-wide license to publish the Supplemental Material files. Please contact the corresponding author directly for reuse.
