# Supplementary material for: Phenotypic screening for metallo-β-lactamases and extended-spectrum β-lactamases in Pseudomonas aeruginosa using the VITEK-2 AST-N443 and Phoenix NMIC-486 cards
Source: Microbiol Spectr. 2026 Sep 23;14(11):e02235-26. doi: 10.1128/spectrum.02235-26 (PMC13729093; doi:10.1128/spectrum.02235-26)
Supplement: Supplementary file 1 [file spectrum.02235-26-s0001.docx]

**Supplementary data**

**Table S1 Genetic features and minimum inhibitory concentrations of the CRPA included in the study**

| Study CRPAZU no. | Origin | ST | Carbapenemase | ESBL | Intrinsic AmpC (PDC) | Intrinsic oxacillinase | Others | Resistotype | VITEK-2 AST-N443 | | | | | | | | | Phoenix NMIC-486 | | | | | | | | | |
| --- | --- | --- | --- | --- | --- | --- | --- | --- | --- | --- | --- | --- | --- | --- | --- | --- | --- | --- | --- | --- | --- | --- | --- | --- | --- | --- | --- |
|  |  |  |  |  |  |  |  |  | **ATM** | **CAZ** | **C/T** | **C/A** | **FEP** | **IMP** | **I/R** | **MEM** | **P/T** | **ATM** | **CAZ** | **C/T** | **C/A** | **FEP** | **IMP** | **I/R** | **MEM** | **M/V** | **P/T** |
| 002 | Switzerland, Zurich | 664 | - | GES-12 | PDC-98 | OXA-50 | - | ESBL | >= 64 | 64 | 32 | 16 | 32 | 16 | 8 | 16 | 128 | >16 | >16 | > 8/4 | 8/4 | >16 | >8 | > 4/4 | 32 | > 16/8 | 64/4 |
| 003 | Switzerland, Zurich | 2572 | - | - | PDC-374 | OXA-488 | - | - | >= 64 | 64 | 32 | 8 | 32 | 16 | 8 | 16 | 128 | >16 | >16 | 4/4 | 2/4 | >16 | >8 | 4/4 | 16 | 8/8 | 16/4 |
| 004 | Switzerland, Zurich | 235 | VIM-4 | - | PDC-35 | OXA-488 | OXA-35/CARB-2 | MBL | 16 | 8 | 32 | 16 | 32 | 16 | 16 | 16 | 128 | 16 | >16 | > 8/4 | > 16/4 | >16 | >8 | > 4/4 | >32 | > 16/8 | > 64/4 |
| 007 | Switzerland, Zurich | 245 | IMP-18 | - | PDC-5 | OXA-494 | OXA-10 | MBL | 4 | 64 | 32 | 16 | 32 | 8 | 8 | 4 | 32 | 8 | >16 | > 8/4 | > 16/4 | >16 | 8 | > 4/4 | 2 | ≤4/8 | > 64/4 |
| 008 | Switzerland, Zurich | 282 | VIM-4 | - | PDC-103 | OXA-50 | CARB-2 | MBL | 16 | 16 | 32 | 16 | 32 | 16 | 16 | 16 | 128 | 16 | >16 | > 8/4 | > 16/4 | >16 | >8 | > 4/4 | >32 | > 16/8 | > 64/4 |
| 009 | Switzerland, Zurich | 357 | IMP-7 | - | PDC-11 | OXA-846 | OXA-2 | MBL | 32 | 64 | 32 | 16 | 32 | 16 | 16 | 16 | 128 | >16 | >16 | > 8/4 | > 8/4 | >16 | >4 | > 4/4 | >32 | > 16/8 | 32/4 |
| 010 | Pakistan | 4936 | IMP-1 | - | PDC-35 | OXA-50 | OXA-10/PAC-1 | MBL | >= 64 | 64 | 32 | 16 | 32 | 16 | 16 | 16 | 128 | >16 | >16 | > 8/4 | > 16/4 | >16 | >8 | > 4/4 | >32 | > 16/8 | > 64/4 |
| 011 | Switzerland, Zurich | 285 | - | - | PDC-1 | OXA-494 | - | - | >= 64 | 64 | 8 | 16 | 32 | 16 | 2 | 16 | 128 | 16 | >16 | 4/4 | > 16/4 | >16 | 8 | 2/4 | 16 | 16/8 | > 64/4 |
| 012 | Switzerland, Zurich | 385 | - | - | PDC-5 | OXA-396 | - | - | 32 | 32 | 2 | 4 | 16 | 16 | 2 | 16 | 128 | >16 | >16 | > 8/4 | 16/4 | >16 | >8 | > 4/4 | 16 | 16/8 | > 64/4 |
| 013 | Switzerland, Zurich | 111 | VIM-2 | - | PDC-3 | OXA-395 | - | MBL | 16 | 16 | 32 | 16 | 16 | 16 | 16 | 16 | 32 | 16 | >16 | > 8/4 | > 16/4 | 16 | >8 | > 4/4 | >32 | > 16/8 | 32/4 |
| 014 | Switzerland, Zurich | 316 | - | - | PDC-36 | OXA-395 | OXA-10 | - | >= 64 | 16 | 8 | 2 | 32 | 16 | 4 | 16 | 128 | >16 | >16 | 8/4 | 4/4 | >16 | >8 | > 4/4 | >32 | > 16/8 | > 64/4 |
| 015 | Switzerland, Zurich | 316 | - | - | PDC-36 | OXA-395 | OXA-10 | - | 32 | 16 | 8 | 2 | 16 | 16 | 4 | 16 | 128 | >16 | >16 | > 8/4 | > 16/4 | >16 | >8 | > 4/4 | >32 | > 16/8 | > 64/4 |
| 016 | Switzerland, Zurich | 3416 | - | - | PDC-23 | OXA-848 | - | - | >= 64 | 16 | 1 | 8 | 8 | 16 | 8 | 16 | 128 | >16 | >16 | 1/4 | 8/4 | >16 | >8 | > 4/4 | 32 | > 16/8 | 32/4 |
| 017 | Pakistan | 4936 | IMP-1 | - | PDC-35 | OXA-50 | OXA-10/PAC-1 | MBL | >= 64 | 64 | 32 | 16 | 32 | 16 | 16 | 16 | 128 | >16 | >16 | > 8/4 | > 16/4 | >16 | >8 | > 4/4 | >32 | > 16/8 | > 64/4 |
| 018 | Switzerland, Zurich | 316 | - | - | PDC-36 | OXA-395 | OXA-10 | - | >= 64 | 64 | 8 | 8 | 32 | 16 | 2 | 16 | 128 | >16 | >16 | 2/4 | 4/4 | >16 | >8 | 2/4 | 16 | 16/8 | > 64/4 |
| 019 | Switzerland, Zurich | 357 | IMP-26/OXA-181 | VEB-9 | PDC-3 | OXA-395 | OXA-21 | MBL+OXA-48+ESBL | >= 64 | 64 | 32 | 16 | 32 | 16 | 16 | 16 | 128 | >16 | >16 | > 8/4 | > 16/4 | >16 | >8 | > 4/4 | >32 | > 16/8 | > 64/4 |
| 020 | Switzerland, Zurich | 3416 | - | - | PDC-23 | OXA-848 | - | - | >= 64 | 16 | 2 | 16 | 16 | 16 | 16 | 16 | 32 | >16 | 16 | 2/4 | > 8/4 | >16 | >4 | > 4/4 | >32 | > 16/8 | 32/4 |
| 021 | Switzerland, Zurich | 111 | VIM-2 | - | PDC-3 | OXA-395 | - | MBL | 16 | 32 | 32 | 16 | 16 | 16 | 16 | 16 | 64 | 16 | >16 | > 8/4 | > 16/4 | >16 | >8 | > 4/4 | >32 | > 16/8 | 64/4 |
| 022 | Switzerland, Zurich | 2572 | - | - | PDC-3 | OXA-488 | - | - | >= 64 | 64 | 4 | 16 | 32 | 16 | 2 | 16 | 128 | >16 | >16 | 2/4 | 4/4 | >16 | >8 | 2/4 | 16 | 16/8 | > 64/4 |
| 024 | Switzerland, Zurich | 664 | - | GES-12 | PDC-98 | OXA-50 | - | ESBL | >= 64 | 64 | 32 | 16 | 32 | 8 | 8 | 16 | 128 | >16 | >16 | > 8/4 | 8/4 | >16 | >8 | > 4/4 | 32 | > 16/8 | 64/4 |
| 025 | Switzerland, Zurich | 111 | VIM-4 | - | PDC-3 | OXA-395 | OXA-9/CARB-2 | MBL | 16 | 32 | 32 | 16 | 32 | 16 | 16 | 16 | 128 | 8 | >16 | > 8/4 | > 8/4 | >16 | >4 | > 4/4 | 32 | > 16/8 | > 64/4 |
| 137 | Switzerland, Zurich | 385 | - | - | PDC-374 | OXA-396 | - | - | >= 64 | 64 | 4 | 4 | 16 | 16 | 2 | 8 | 128 | 16 | >16 | 2/4 | 2/4 | >16 | >8 | ≤1/4 | 8 | ≤4/8 | > 64/4 |
| 028 | Switzerland, Zurich | 235 | VIM-2 | - | PDC-35 | OXA-488 | - | MBL | 4 | 64 | 32 | 16 | 32 | 16 | 16 | 16 | 128 | 8 | >16 | > 8/4 | > 16/4 | >16 | >8 | > 4/4 | >32 | > 16/8 | 64/4 |
| 029 | Switzerland, Zurich | 111 | VIM-2 | - | PDC-3 | OXA-395 | - | MBL | 16 | 16 | 32 | 16 | 16 | 16 | 16 | 16 | 32 | 16 | >16 | > 8/4 | > 16/4 | >16 | >8 | > 4/4 | >32 | > 16/8 | >32/4 |
| 030 | Switzerland, Zurich | 1047 | IMP-1 | - | PDC-12 | OXA-488 | OXA-10 | MBL | >= 64 | 64 | 32 | 16 | 32 | 16 | 16 | 16 | 128 | >16 | >16 | > 8/4 | > 16/4 | >16 | >8 | > 4/4 | >32 | > 16/8 | > 64/4 |
| 031 | Switzerland, Zurich | 111 | - | - | PDC-3 | OXA-395 | OXA-14/CARB-2 | - | 32 | 64 | 32 | 16 | 32 | 16 | 8 | 16 | 128 | >16 | >16 | > 8/4 | > 8/4 | >16 | >4 | 4/4 | 32 | > 16/8 | > 64/4 |
| 032 | Switzerland, Zurich | 155 | VIM-1 | GES-7 | PDC-5 | OXA-396 | - | MBL+ESBL | >= 64 | 64 | 32 | 16 | 32 | 16 | 16 | 8 | 128 | >16 | >16 | > 8/4 | > 8/4 | >16 | >4 | > 4/4 | 16 | 16/8 | > 64/4 |
| 033 | Switzerland, Zurich | 988 | - | - | PDC-23 | OXA-904 | - | - | >= 64 | 64 | 32 | 16 | 32 | 16 | 8 | 16 | 128 | 2 | >16 | 4/4 | 8/4 | >16 | >4 | > 4/4 | 32 | > 16/8 | > 64/4 |
| 034 | Switzerland, Zurich | 274 | - | - | PDC-24 | OXA-486 | - | - | >= 64 | 64 | 32 | 16 | 32 | 16 | 8 | 16 | 128 | >16 | >16 | > 8/4 | > 8/4 | >16 | >4 | > 4/4 | >32 | > 16/8 | > 64/4 |
| 035 | Switzerland, Zurich | 253 | - | - | PDC-34 | OXA-488 | - | - | 16 | 2 | 1 | 2 | 2 | 16 | 2 | 8 | 8 | 16 |  | 4/4 | 8/4 | 8 | >8 | > 4/4 |  | 16/8 | > 64/4 |
| 036 | Switzerland, Zurich | 111 | VIM-2 | - | PDC-3 | OXA-395 | - | MBL | 16 | 16 | 32 | 16 | 16 | 16 | 16 | 16 | 128 | 16 | >16 | > 8/4 | > 16/4 | >16 | >8 | > 4/4 | >32 | > 16/8 | 64/4 |
| 037 | Switzerland, Zurich | 4072 | - | - | PDC-374 | OXA-486 | - | - | >= 64 | 64 | 32 | 16 | 32 | 16 | 16 | 16 | 128 | >16 | >16 | > 8/4 | > 16/4 | >16 | >8 | > 4/4 | >32 | > 16/8 | > 64/4 |
| 038 | Switzerland, Zurich | 235 | VIM-4 | - | PDC-35 | OXA-488 | OXA-35/CARB-2 | MBL | 32 | 32 | 32 | 16 | 32 | 16 | 16 | 16 | 128 | >16 | >16 | > 8/4 | > 16/4 | >16 | >8 | > 4/4 | >32 | 16/8 | > 64/4 |
| 039 | Switzerland, Zurich | 1420 | - | - | PDC-19a | OXA-395 | - | - | >= 64 | 32 | 2 | 16 | 16 | 16 | 4 | 16 | 128 | >16 | >16 | 1/4 | 8/4 | >16 | >8 | 2/4 | 32 | > 16/8 | >32/4 |
| 040 | Switzerland, Zurich | 485 | - | - | PDC-6 | OXA-50 | - | - | INTERR | INTERR | 8 | 16 | 16 | 16 | 16 | INTERR | 128 | >16 | >16 | 8/4 | 8/4 | >16 | >4 | > 4/4 | 32 | > 16/8 | > 64/4 |
| 041 | Switzerland, Zurich | 235 | VIM-4 | - | PDC-35 | OXA-488 | CARB-2 | MBL | 4 | 64 | 32 | 16 | 32 | 16 | 16 | 16 | 128 | 4 | >16 | > 8/4 | > 8/4 | >16 | >4 | > 4/4 | >32 | > 16/8 | > 64/4 |
| 044 | Switzerland, Zurich | n.t. | - | - | PDC-432 | OXA-1124 | - | - | 16 | 8 | 4 | 4 | 32 | 16 | 16 | 8 | INTERR | 8 | 16 | 4/4 | 4/4 | >16 | >4 | > 4/4 | >32 | > 16/8 | 16/4 |
| 045 | Switzerland, Zurich | 235 | - | VEB-16 | PDC-35 | OXA-488 | OXA-10 | ESBL | >= 64 | 64 | 32 | 16 | 32 | 16 | 16 | 16 | 128 | >16 | >16 | > 8/4 | > 8/4 | >16 | >4 | 4/4 | 32 | > 16/8 | > 64/4 |
| 046 | Switzerland, Zurich | 235 | VIM-4 | - | PDC-35 | OXA-488 | OXA-35/CARB-2 | MBL | 32 | 64 | 32 | 16 | 32 | 16 | 16 | 16 | 128 | 16 | >16 | > 8/4 | > 8/4 | >16 | >4 | > 4/4 | >32 | > 16/8 | > 64/4 |
| 047 | Switzerland, Zurich | 111 | - | - | PDC-3 | OXA-395 | OXA-9 | - | 32 | 2 | 16 | 16 | 16 | 16 | 16 | 16 | 128 | >16 | >16 | 8/4 | 2/4 | >16 | >4 | > 4/4 | 32 | 16/8 | 64/4 |
| 138 | Switzerland, Zurich | 111 | - | - | PDC-55 | OXA-395 | OXA-9 | - | 16 | 8 | 1 | 4 | 8 | 16 | 4 | 16 | 128 | 16 | 16 | ≤1/4 | 4/4 | 8 | >4 | 2/4 | 16 | 16/8 | 32/4 |
| 048 | Switzerland, Zurich | 357 | - | VEB-9 | PDC-11 | OXA-846 | OXA-10 | ESBL | >= 64 | 64 | 32 | 16 | 32 | 16 | 4 | 16 | 128 | >16 | >16 | > 8/4 | > 8/4 | >16 | >4 | 4/4 | 32 | > 16/8 | 64/4 |
| 049 | Switzerland, Zurich | 235 | AIM-1 | SHV-5 | PDC-35 | OXA-488 | - | MBL+ESBL | >= 64 | 64 | 32 | 16 | 32 | 16 | 16 | 16 | 128 | >16 | >16 | > 8/4 | > 8/4 | >16 | >4 | > 4/4 | >32 | > 16/8 | 64/4 |
| 050 | Switzerland, Zurich | 235 | VIM-28 | SHV-2a | PDC-35 | OXA-488 | - | MBL+ESBL | >= 64 | 64 | 32 | 16 | 32 | 16 | 16 | 16 | 128 | >16 | >16 | > 8/4 | > 8/4 | >16 | >4 | > 4/4 | >32 | > 16/8 | > 64/4 |
| 051 | Switzerland, Zurich | 111 | VIM-4 | - | PDC-3 | OXA-395 | OXA-9/CARBA-2 | MBL | 16 | 64 | 32 | 16 | 32 | 16 | 16 | 16 | 128 | 16 | >16 | > 8/4 | > 8/4 | >16 | >4 | > 4/4 | >32 | > 16/8 | > 64/4 |
| 052 | Switzerland, Zurich | 111 | - | - | PDC-3 | OXA-395 | OXA-9 | - | >= 64 | 64 | 4 | 16 | 32 | 16 | 16 | 16 | 128 | >16 | >16 | 4/4 | 8/4 | >16 | >4 | 2/4 | 32 | 16/8 | > 64/4 |
| 053 | Switzerland, Zurich | 235 | VIM-2 | - | PDC-35 | OXA-488 | - | MBL | 32 | 64 | 32 | 16 | 32 | 16 | 16 | 16 | 128 | >16 | >16 | > 8/4 | > 8/4 | >16 | >4 | > 4/4 | >32 | > 16/8 | > 64/4 |
| 054 | Switzerland, Zurich | 235 | VIM-4 | - | PDC-35 | OXA-488 | OXA-35/CARB-2 | MBL | 32 | 64 | 32 | 16 | 32 | 16 | 16 | 16 | 128 | >16 | >16 | > 8/4 | > 8/4 | >16 | >4 | > 4/4 | >32 | > 16/8 | > 64/4 |
| 055 | Switzerland, Zurich | 235 | VIM-4 | - | PDC-35 | OXA-488 | OXA-35/CARB-2 | MBL | >= 64 | 64 | 32 | 16 | 32 | 16 | 16 | 16 | 128 | 16 | >16 | > 8/4 | > 8/4 | >16 | >4 | > 4/4 | >32 | > 16/8 | > 64/4 |
| 056 | Switzerland, Zurich | 175 | VIM-1 | - | PDC-1 | OXA-50 | - | MBL | 4 | 64 | 32 | 16 | 32 | 16 | 16 | 16 | 128 | 8 | >16 | > 8/4 | > 8/4 | >16 | >4 | > 4/4 | >32 | > 16/8 | > 64/4 |
| 057 | Switzerland, Zurich | 235 | - | - | PDC-35 | OXA-488 | OXA-35/CARB-2 | - | >= 64 | 16 | 8 | 8 | 32 | 16 | 8 | 16 | 128 | >16 | 16 | ≤1/4 | > 8/4 | 2 | ≤0.25 | ≤1/4 | ≤0.5 | ≤4/8 | ≤4/4 |
| 058 | Switzerland, Zurich | 164 | - | - | PDC-121 | OXA-486 | - | - | >= 64 | 16 | 1 | 16 | 16 | 16 | 2 | 16 | 32 | >16 | 16 | 2/4 | 8/4 | >16 | >4 | 2/4 | >32 | > 16/8 | 14/4 |
| 059 | Switzerland, Zurich | 357 | - | VEB-9 | PDC-11 | OXA-846 | OXA-10 | ESBL | >= 64 | 64 | 32 | 16 | 32 | 16 | 16 | 16 | 128 | >16 | >16 | > 8/4 | > 8/4 | >16 | >4 | 4/4 | 32 | > 16/8 | > 64/4 |
| 060 | Switzerland, Zurich | 235 | VIM-4 | - | PDC-35 | OXA-488 | OXA-35/CARB-2 | MBL | 32 | 32 | 32 | 16 | 32 | 16 | 16 | 16 | 128 | >16 | >16 | > 8/4 | > 8/4 | >16 | >4 | > 4/4 | >32 | > 16/8 | > 64/4 |
| 061 | Switzerland, Zurich | 235 | - | GES-52 | PDC-35 | OXA-488 | - | ESBL | >= 64 | 64 | 32 | 16 | 32 | 16 | 4 | 16 | 64 | >16 | >16 | > 8/4 | > 8/4 | >16 | >4 | 4/4 | 16 | 16/8 | 32/4 |
| 062 | Switzerland, Zurich | 635 | - | - | PDC-8 | OXA-395 | - | - | >= 64 | 64 | 8 | 16 | 32 | 16 | 2 | 16 | 128 | >16 | >16 | 8/4 | > 8/4 | >16 | >4 | 2/4 | >32 | > 16/8 | > 64/4 |
| 063 | Switzerland, Zurich | 235 | VIM-2 | - | PDC-35 | OXA-488 | OXA-10 | MBL | 16 | 16 | 32 | 16 | 16 | 16 | 16 | 16 | 128 | >16 | >16 | > 8/4 | > 8/4 | >16 | >4 | > 4/4 | >32 | > 16/8 | > 64/4 |
| 064 | Switzerland, Zurich | 316 | - | - | PDC-36 | OXA-395 | OXA-10 | - | >= 64 | 64 | 8 | 8 | 32 | 16 | 8 | 16 | 128 | >16 | >16 | > 8/4 | > 8/4 | >16 | >4 | > 4/4 | >32 | > 16/8 | > 64/4 |
| 065 | Switzerland, Zurich | 316 | - | - | PDC-36 | OXA-395 | OXA-10 | - | 16 | 16 | 4 | 2 | 16 | 16 | 4 | 16 | 128 | 16 | 16 | 4/4 | 4/4 | >16 | >4 | 4/4 | 16 | 16/8 | > 64/4 |
| 066 | Switzerland, Zurich | 111 | VIM-2 | - | PDC-3 | OXA-395 | - | MBL | 16 | 32 | 32 | 16 | 16 | 16 | 16 | 16 | INTERR | 16 | >16 | > 8/4 | > 8/4 | >16 | >4 | > 4/4 | >32 | > 16/8 | 32/4 |
| 067 | Switzerland, Zurich | 235 | NDM-1/VIM-2 | - | PDC-35 | OXA-488 | - | MBL | 2 | 64 | 32 | 16 | 32 | 16 | 16 | 16 | 128 | 8 | >16 | > 8/4 | > 8/4 | >16 | >4 | > 4/4 | >32 | > 16/8 | 64/4 |
| 068 | Switzerland, Zurich | 2553 | IMP-1 | - | PDC-3 | OXA-486 | - | MBL | 16 | 64 | 32 | 16 | 32 | 16 | 16 | 16 | 128 | ≤1 | >16 | > 8/4 | > 8/4 | >16 | >4 | > 4/4 | >32 | 16/8 | 32/4 |
| 069 | Switzerland, Zurich | 155 | - | - | PDC-5 | OXA-396 | - | - | >= 64 | 64 | 4 | 16 | 32 | 8 | 2 | 16 | 128 | >16 | >16 | 4/4 | > 8/4 | >16 | >4 | 4/4 | 32 | > 16/8 | > 64/4 |
| 070 | Switzerland, Zurich | 266 | - | - | PDC-1 | OXA-486 | - | - | >= 64 | 64 | 2 | 4 | 16 | 16 | 1 | 8 | 128 | >16 | >16 | 4/4 | 8/4 | 16 | >4 | 4/4 | 8 | 8/8 | > 64/4 |
| 071 | Switzerland, Zurich | 357 | VIM-5 | VEB-14 | PDC-11 | OXA-846 | OXA-10 | MBL+ESBL | >= 64 | 64 | 32 | 16 | 32 | 16 | 16 | 16 | 128 | >16 | >16 | > 8/4 | > 8/4 | >16 | >4 | > 4/4 | >32 | > 16/8 | 32/4 |
| 072 | Switzerland, Zurich | 1047 | IMP-1 | - | PDC-12 | OXA-488 | OXA-10 | MBL | >= 64 | 64 | 32 | 16 | 32 | 16 | 8 | 16 | 128 | >16 | >16 | > 8/4 | > 8/4 | >16 | >4 | > 4/4 | >32 | > 16/8 | > 64/4 |
| 073 | Switzerland, Zurich | 235 | VIM-4 | - | PDC-35 | OXA-488 | CARB-2 | MBL | 2 | 64 | 32 | 16 | 32 | 16 | 16 | 16 | 128 | 4 | >16 | > 8/4 | > 8/4 | >16 | >4 | > 4/4 | >32 | > 16/8 | > 64/4 |
| 074 | Switzerland, Zurich | 654 | VIM-2 | - | PDC-3 | OXA-396 | - | MBL | 32 | 64 | 32 | 16 | 32 | 16 | 16 | 16 | 128 | 16 | >16 | > 8/4 | > 8/4 | >16 | >4 | > 4/4 | >32 | > 16/8 | 64/4 |
| 075 | Switzerland, Zurich | 179 | - | PER-1 | PDC-8 | OXA-396 | OXA-4 | ESBL | >= 64 | 64 | 32 | 16 | 32 | 16 | 16 | 16 | 128 | >16 | >16 | > 8/4 | > 8/4 | >16 | >4 | 4/4 | 32 | > 16/8 | > 64/4 |
| 076 | Switzerland, Zurich | 773 | NDM-1 | - | PDC-16 | OXA-395 | - | MBL | 16 | 64 | 32 | 16 | 32 | 16 | 16 | 16 | 128 | 16 | >16 | > 8/4 | > 8/4 | >16 | >4 | > 4/4 | >32 | > 16/8 | > 64/4 |
| 077 | Switzerland, Zurich | 233 | VIM-5 | - | PDC-3 | OXA-486 | OXA-4 | MBL | 16 | 32 | 32 | 16 | 32 | 16 | 16 | 16 | 128 | 8 | >16 | > 8/4 | > 8/4 | >16 | >4 | > 4/4 | >32 | > 16/8 | > 64/4 |
| 078 | Switzerland, Zurich | 773 | NDM-1 | - | PDC-16 | OXA-395 | - | MBL | 4 | 64 | 32 | 16 | 32 | 16 | 16 | 16 | 128 | 4 | >16 | > 8/4 | > 8/4 | >16 | >4 | > 4/4 | >32 | > 16/8 | > 64/4 |
| 079 | Switzerland, Zurich | 316 | VIM-2 | - | PDC-36 | OXA-395 | OXA-10/OXA-796 | MBL | 16 | 32 | 32 | 16 | 32 | 16 | 16 | 16 | 128 | 16 | >16 | > 8/4 | > 8/4 | >16 | >4 | > 4/4 | >32 | > 16/8 | > 64/4 |
| 080 | Switzerland, Zurich | 654 | VIM-2 | - | PDC-3 | OXA-396 | - | MBL | 32 | 64 | 32 | 16 | 32 | 16 | 16 | 16 | 128 | 16 | >16 | > 8/4 | > 8/4 | >16 | >4 | > 4/4 | >32 | > 16/8 | 64/4 |
| 081 | Switzerland, Zurich | 1047 | IMP-1 | - | PDC-12 | OXA-488 | OXA-10 | MBL | 32 | 64 | 32 | 16 | 32 | 16 | 16 | 16 | 128 | >16 | >16 | > 8/4 | > 8/4 | >16 | >4 | > 4/4 | >32 | > 16/8 | > 64/4 |
| 082 | Pakistan | 4936 | IMP-1 | - | PDC-35 | OXA-50 | OXA-10/PAC-1 | MBL | >= 64 | 64 | 32 | 16 | 32 | 16 | 16 | 16 | 128 | >16 | >16 | > 8/4 | > 8/4 | >16 | >4 | > 4/4 | >32 | > 16/8 | > 64/4 |
| 083 | Switzerland, Zurich | 1047 | IMP-1 |  | PDC-12 | OXA-488 | OXA-10 | MBL | >= 64 | 64 | 32 | 16 | 32 | 16 | 16 | 16 | 128 | >16 | >16 | > 8/4 | > 8/4 | >16 | >4 | > 4/4 | >32 | > 16/8 | > 64/4 |
| 084 | Switzerland, Zurich | 235 | NDM-1 | PME-1 | PDC-35 | OXA-488 | - | MBL+ESBL | >= 64 | 64 | 32 | 16 | 32 | 16 | 2 | 16 | 128 | >16 | >16 | > 8/4 | > 8/4 | >16 | 4 | 2/4 | 32 | > 16/8 | > 64/4 |
| 085 | Switzerland, Zurich | 2167 | - | - | PDC-309 | OXA-1125 | - | - | >= 64 | 64 | 32 | 16 | 32 | 4 | 4 | 16 | 128 | >16 | >16 | > 8/4 | > 8/4 | 16 | >4 | > 4/4 | >32 | > 16/8 | 64/4 |
| 139 | Switzerland, Zurich | 255 | - | - | PDC-55 | OXA-905 | - | - | >= 64 | 64 | 32 | 16 | 32 | 1 | 0,5 | 1 | 128 | >16 | >16 | > 8/4 | > 8/4 | >16 | 1 | ≤1/4 | 2 | ≤4/8 | 64/4 |
| 086 | Switzerland, Zurich | 244 | NDM-1 | PME-1 | PDC-1 | OXA-847 | OXA-10 | MBL+ESBL | >= 64 | 64 | 32 | 16 | 32 | 16 | 16 | 16 | 128 | >16 | >16 | > 8/4 | > 8/4 | >16 | >4 | > 4/4 | >32 | > 16/8 | > 64/4 |
| 087 | Switzerland, Zurich | 244 | NDM-1 | PME-1 | PDC-374 | OXA-847 | OXA-10 | MBL+ESBL | >= 64 | 64 | 32 | 16 | 32 | 16 | 16 | 16 | 128 | >16 | >16 | > 8/4 | > 8/4 | >16 | >4 | > 4/4 | >32 | > 16/8 | > 64/4 |
| 139 | Switzerland, Zurich | 255 | - | - | PDC-55 | OXA-905 | - | - | >= 64 | 64 | 32 | 16 | 32 | 1 | 0,5 | 1 | 128 | >16 | >16 | > 8/4 | > 8/4 | >16 | 1 | ≤1/4 | 2 | ≤4/8 | 64/4 |
| 139 | Switzerland, Zurich | 255 | - | - | PDC-55 | OXA-905 | - | - | >= 64 | 64 | 32 | 16 | 32 | 1 | 0,5 | 1 | 128 | >16 | >16 | > 8/4 | > 8/4 | >16 | 1 | ≤1/4 | 2 | ≤4/8 | 64/4 |
| 088 | Switzerland, Zurich | 235 | IMP-1 | - | PDC-35 | OXA-488 | - | MBL | 32 | 64 | 32 | 16 | 32 | 8 | 16 | 16 | 32 | >16 | >16 | > 8/4 | > 8/4 | >16 | >4 | 4/4 | >32 | > 16/8 | 32/4 |
| 089 | Switzerland, Zurich | 664 | VIM-4 | - | PDC-98 | OXA-50 | - | MBL | 16 | 64 | 32 | 16 | 32 | 16 | 16 | 16 | 128 | >16 | >16 | > 8/4 | > 8/4 | >16 | >4 | > 4/4 | >32 | > 16/8 | > 64/4 |
| 091 | Pakistan | 4936 | IMP-1 | - | PDC-35 | OXA-50 | OXA-10/PAC-1 | MBL | >= 64 | 64 | 32 | 16 | 32 | 16 | 16 | 16 | 128 | >16 | >16 | > 8/4 | > 8/4 | >16 | >4 | > 4/4 | >32 | > 16/8 | > 64/4 |
| 092 | Pakistan | 4936 | IMP-1 | - | PDC-35 | OXA-50 | OXA-10/PAC-1 | MBL | >= 64 | 64 | 32 | 16 | 32 | 16 | 16 | 16 | 128 | >16 | >16 | > 8/4 | > 8/4 | >16 | >4 | > 4/4 | >32 | > 16/8 | > 64/4 |
| 093 | Pakistan | 4936 | IMP-1 | - | PDC-35 | OXA-50 | OXA-10/PAC-1 | MBL | >= 64 | 64 | 32 | 16 | 32 | 16 | 16 | 16 | 128 | >16 | >16 | > 8/4 | > 8/4 | >16 | >4 | > 4/4 | >32 | > 16/8 | > 64/4 |
| 094 | Pakistan | 1047 | - | GES-13 | PDC-12 | OXA-488 | OXA-10 | ESBL | >= 64 | 64 | 8 | 16 | 32 | 16 | 8 | 16 | 128 | >16 | >16 | > 8/4 | > 8/4 | >16 | >4 | 4/4 | >32 | > 16/8 | > 64/4 |
| 095 | Pakistan | 1047 | - | GES-13 | PDC-12 | OXA-488 | OXA-10 | ESBL | >= 64 | 64 | 32 | 16 | 32 | 16 | 16 | 16 | 128 | >16 | >16 | > 8/4 | 8/4 | >16 | >4 | > 4/4 | >32 | > 16/8 | > 64/4 |
| 096 | Pakistan | 235 | NDM-1/IMP-1 | - | PDC-35 | OXA-488 | OXA-101 | MBL | >= 64 | 64 | 32 | 16 | 32 | 16 | 16 | 16 | 128 | >16 | >16 | > 8/4 | > 8/4 | >16 | >4 | > 4/4 | >32 | > 16/8 | 64/4 |
| 097 | Pakistan | 4936 | IMP-1 | - | PDC-35 | OXA-50 | OXA-10/PAC-1 | MBL | >= 64 | 64 | 32 | 16 | 32 | 16 | 16 | 16 | 128 | >16 | >16 | > 8/4 | > 8/4 | >16 | >4 | > 4/4 | >32 | > 16/8 | 32/4 |
| 098 | Pakistan | 235 | NDM-1/IMP-1 | - | PDC-35 | OXA-488 | OXA-101 | MBL | >= 64 | 64 | 32 | 16 | 32 | 16 | 16 | 16 | 128 | >16 | >16 | > 8/4 | > 8/4 | >16 | >4 | > 4/4 | >32 | > 16/8 | > 64/4 |
| 099 | Pakistan | 4936 | IMP-1 | - | PDC-35 | OXA-50 | OXA-10/PAC-1 | MBL | >= 64 | 64 | 32 | 16 | 32 | 16 | 16 | 16 | 128 | >16 | >16 | > 8/4 | > 8/4 | >16 | >4 | > 4/4 | >32 | > 16/8 | > 64/4 |
| 100 | Pakistan | 654 | NDM-1 |  | PDC-3 | OXA-396 | - | MBL | 16 | 64 | 32 | 16 | 32 | 16 | 16 | 16 | 128 | 16 | >16 | > 8/4 | > 8/4 | >16 | >4 | > 4/4 | >32 | > 16/8 | > 64/4 |
| 101 | Switzerland, Zurich | 395 | - | - | PDC-8 | OXA-905 | - | - | >= 64 | 16 | 4 | 16 | 32 | 16 | 16 | 16 | 128 | 16 | >16 | 2/4 | > 8/4 | >16 | >4 | > 4/4 | >32 | > 16/8 | > 64/4 |
| 102 | Switzerland, Zurich | 235 | VIM-4 | - | PDC-35 | OXA-488 | OXA-35/CARB-2 | MBL | >= 64 | 64 | 32 | 16 | 32 | 16 | 16 | 16 | 128 | >16 | >16 | > 8/4 | > 8/4 | >16 | >4 | > 4/4 | >32 | > 16/8 | > 64/4 |
| 103 | WRAIR, USA | 317 | - | - | PDC-16 | OXA-488 | - | - | >= 64 | 64 | 8 | 8 | 32 | 16 | 2 | 8 | 128 | >16 | >16 | 2/4 | 2/4 | >16 | >4 | 4/4 | >32 | > 16/8 | 64/4 |
| 104 | WRAIR, USA | 5519 | - | - | PDC-35 | OXA-488 | - | - | 16 | 16 | 4 | 8 | 16 | 16 | 16 | 16 | 128 | >16 | >16 | 2/4 | 4/4 | 16 | >4 | 4/4 | 16 | 16/8 | > 64/4 |
| 105 | WRAIR, USA | 6220 | VIM-6 | - | PDC-1 | OXA-847 | OXA-10 | MBL | >= 64 | 64 | 32 | 16 | 32 | 16 | 16 | 16 | 128 | 4 | >16 | > 8/4 | > 8/4 | 8 | >4 | > 4/4 | >32 | > 16/8 | > 64/4 |
| 106 | WRAIR, USA | 6241 | - | PER-1 | PDC-62 | OXA-395 | OXA-2 | ESBL | >= 64 | 64 | 32 | 16 | 32 | 16 | 8 | 16 | 128 | >16 | >16 | > 8/4 | > 8/4 | >16 | >4 | 4/4 | 32 | 16/8 | 64/4 |
| 107 | WRAIR, USA | 6678 | - | - | PDC-35 | OXA-488 | OXA-2 | - | >= 64 | 64 | 4 | 16 | 16 | 16 | 16 | 16 | 128 | >16 | >16 | 2/4 | 8/4 | 16 | >4 | 4/4 | 32 | > 16/8 | > 64/4 |
| 108 | WRAIR, USA | 8141 | - | PER-1 | PDC-33/62 | OXA-395 | OXA-2 | ESBL | >= 64 | 64 | 32 | 16 | 32 | 16 | 8 | 16 | 128 | >16 | >16 | > 8/4 | > 8/4 | >16 | >4 | 4/4 | 32 | > 16/8 | 64/4 |
| 109 | WRAIR, USA | 8914 | - | - | PDC-3 | OXA-395 | OXA-10 | - | 16 | 16 | 4 | 2 | 16 | 16 | 16 | 16 | 128 |  | 16 | 4/4 | 2/4 | >16 | >4 | 4/4 | 16 | 16/8 | > 64/4 |
| 110 | WRAIR, USA | 23861 | KPC-2 | - | PDC-103 | OXA-50 | OXA-2/TEM-1A | KPC | >= 64 | 16 | 8 | 2 | 32 | 16 | 8 | 16 | 128 | >16 | 16 | 4/4 | 1/4 | >16 | >4 | > 4/4 | >32 | > 16/8 | 64/4 |
| 111 | WRAIR, USA | 12914 | - | VEB-1 | PDC-11 | OXA-846 | OXA-10 | ESBL | >= 64 | 64 | 32 | 16 | 32 | 16 | 16 | 16 | 128 | >16 | >16 | > 8/4 | > 8/4 | >16 | >4 | 4/4 | 32 | > 16/8 | > 64/4 |
| 112 | WRAIR, USA | 20176 | VIM-11 | - | PDC-36 | OXA-395 | OXA-2 | MBL | 16 | 64 | 32 | 16 | 32 | 16 | 16 | 1 | 128 | 8 | >16 | > 8/4 | > 8/4 | >16 | >4 | > 4/4 | 2 | ≤4/8 | > 64/4 |
| 114 | Switzerland, Zurich | 111 | VIM-2 | - | PDC-3 | OXA-395 |  | MBL | 16 | 32 | 32 | 16 | 16 | 16 | 16 | 16 | 64 | 4 | >16 | > 8/4 | > 8/4 | >16 | >4 | 4/4 | >32 | > 16/8 | 64/4 |
| 115 | Switzerland, Basel | 111 | VIM-2 | - | PDC-3 | OXA-395 |  | MBL | 16 | 32 | 32 | 16 | 16 | 16 | 16 | 4 | 128 | 8 | >16 | > 8/4 | > 8/4 | 16 | >4 | > 4/4 | 4 | 4/8 | 32/4 |
| 116 | Switzerland, Basel | 233 | VIM-2 | - | PDC-3 | OXA-486 |  | MBL | 8 | 32 | 32 | 16 | 32 | 16 | 16 | 16 | 128 | 8 | >16 | > 8/4 | > 8/4 | >16 | >4 | > 4/4 | 16 | 16/8 | > 64/4 |
| 117 | Switzerland, Basel | 235 | VIM-2 | - | PDC-35 | OXA-488 |  | MBL | 2 | 16 | 32 | 16 | 16 | 16 | 16 | 16 | 128 | 8 | >16 | > 8/4 | > 8/4 | 16 | >4 | > 4/4 | >32 | > 16/8 | > 64/4 |
| 118 | Switzerland, Basel | 3511 | VIM-2 | - | PDC-3 | OXA-494 |  | MBL | >= 64 | 32 | 32 | 16 | 32 | 16 | 16 | 16 | 128 | >16 | >16 | > 8/4 | > 8/4 | >16 | >4 | > 4/4 | >32 | > 16/8 | > 64/4 |
| 119 | Switzerland, Basel | 235 | GES-5 | - | PDC-35 | OXA-488 |  | GES-carba | 16 | 16 | 4 | 4 | 8 | 16 | 16 | 16 | 128 | >16 | 16 | 8/4 | 8/4 | 16 | >4 | > 4/4 | >32 | > 16/8 | 64/4 |
| 120 | Switzerland, Basel | 235 | VIM-1 | - | PDC-35 | OXA-488 |  | MBL | 16 | 64 | 32 | 16 | 32 | 16 | 16 | 16 | 128 | 16 | >16 | > 8/4 | > 8/4 | >16 | >4 | > 4/4 | >32 | > 16/8 | > 64/4 |
| 121 | Switzerland, Basel | 654 | NDM-1/GES-5 | - | PDC-3 | OXA-396 |  | MBL+GES-carba | 32 | 64 | 32 | 16 | 32 | 16 | 16 | 16 | 128 | 16 | >16 | > 8/4 | > 8/4 | >16 | >4 | > 4/4 | >32 | > 16/8 | > 64/4 |
| 122 | Switzerland, Basel | 773 | NDM-1 | - | PDC-16 | OXA-395 | rmtB4 | MBL | 4 | 64 | 32 | 16 | 32 | 16 | 16 | 16 | 128 | 8 | >16 | > 8/4 | > 8/4 | >16 | >4 | > 4/4 | >32 | > 16/8 | > 64/4 |
| 123 | Switzerland, Basel | 773 | NDM-1 | - | PDC-16 | OXA-395 | rmtB4 | MBL | 4 | 64 | 32 | 16 | 32 | 16 | 16 | 16 | 128 | 8 | >16 | > 8/4 | > 8/4 | >16 | >4 | > 4/4 | >32 | > 16/8 | > 64/4 |
| 124 | Switzerland, Basel | 155 | IMP-15 | - | PDC-374 | OXA-396 | OXA-10 | MBL | 8 | 64 | 32 | 16 | 32 | 16 | 2 | 4 | 128 | 16 | >16 | > 8/4 | > 8/4 | >16 | >4 | > 4/4 | 8 | 8/8 | > 64/4 |
| 125 | Switzerland, Basel | 277 | SPM-1 | - | PDC-374 | OXA-494 | rmtD1 | MBL | 16 | 64 | 32 | 16 | 32 | 16 | 16 | 16 | 128 | >16 | >16 | > 8/4 | > 8/4 | >16 | >4 | > 4/4 | >32 | > 16/8 | > 64/4 |
| 126 | Switzerland, Basel | 1109 | - | - | PDC-3 | OXA-494 | - | - | >= 64 | 64 | 4 | 16 | 32 | 16 | 2 | 16 | 128 | >16 | >16 | 2/4 | 4/4 | >16 | >4 | 2/4 | 16 | 8/8 | > 64/4 |
| 127 | Switzerland, Basel | 2351 | - | - | PDC-5 | OXA-50-like (new) | - | - | >= 64 | 64 | 4 | 16 | 32 | 16 | 4 | 16 | 128 | >16 | >16 | 4/4 | > 8/4 | >16 | >4 | > 4/4 | >32 | > 16/8 | > 64/4 |
| 128 | Switzerland, Basel | 2351 | - | - | PDC-5 | OXA-50-like (new) | - | - | >= 64 | 32 | 4 | 16 | 32 | 16 | 4 | 16 | 128 | 16 | >16 | 4/4 | > 8/4 | >16 | >4 | 4/4 | 32 | > 16/8 | > 64/4 |
| 129 | Switzerland, Basel | 2351 | - | - | PDC-5 | OXA-50-like (new) | - | - | >= 64 | 32 | 1 | 16 | 16 | 16 | 8 | 16 | 128 | >16 | >16 | 4/4 | > 8/4 | >16 | >4 | > 4/4 | >32 | > 16/8 | > 64/4 |
| 130 | Switzerland, Basel | 2351 | - | - | PDC-5 | OXA-50-like (new) | - | - | >= 64 | 64 | 4 | 16 | 16 | 16 | 2 | 16 | 128 | >16 | >16 | 4/4 | 8/4 | >16 | >4 | 4/4 | 32 | > 16/8 | > 64/4 |
| 131 | Switzerland, Basel | 2351 | - | - | PDC-5 | OXA-50-like (new) | - | - | >= 64 | 64 | 4 | 16 | 32 | 16 | 2 | 16 | 128 | >16 | >16 | 4/4 | > 8/4 | >16 | >4 | 2/4 | 32 | > 16/8 | > 64/4 |
| 132 | Switzerland, Basel | 3321 | - | - | PDC-5 | OXA-914 | - | - | >= 64 | 64 | 4 | 8 | 32 | 16 | 0,5 | 16 | 128 | >16 | >16 | 4/4 | 4/4 | 16 | >4 | ≤1/4 | 8 | ≤4/8 | > 64/4 |
| 133 | Switzerland, Basel | 1109 | - | - | PDC-3 | OXA-494 | - | - | >= 64 | 64 | 4 | 8 | 32 | 16 | 2 | 16 | 128 | >16 | >16 | 2/4 | 4/4 | >16 | >4 | 2/4 | 16 | 8/8 | > 64/4 |
| 134 | Switzerland, Basel | 244 | - | - | PDC-5 | OXA-847 | - | - | >= 64 | 64 | 2 | 2 | 8 | 16 | 0,5 | 8 | 128 | >16 | >16 | 2/4 | 4/4 | 16 | >4 | ≤1/4 | 8 | ≤4/8 | > 64/4 |
| 135 | Switzerland, Basel | 207 | - | - | PDC-30 | OXA-395 | - | - | >= 64 | 32 | 2 | 16 | 16 | 16 | 2 | 16 | 128 | 4 | >16 | 2/4 | 8/4 | 16 | >4 | 4/4 | 32 | > 16/8 | > 64/4 |
| 136 | Switzerland, Basel | 207 | - | - | PDC-30 | OXA-395 | - | - | >= 64 | 32 | 4 | 16 | 16 | 16 | 16 | 16 | 128 | >16 | >16 | 4/4 | > 8/4 | >16 | >4 | > 4/4 | >32 | > 16/8 | > 64/4 |
| 140 | Pakistan | 4936 | IMP-1 | - | PDC-35 | OXA-50 | OXA-10/PAC-1, rmtF2 | MBL | >= 64 | 64 | 32 | 16 | 32 | 16 | 16 | 16 | 128 | >16 | >16 | > 8/4 | > 8/4 | >16 | >4 | > 4/4 | >32 | > 16/8 | > 64/4 |
| 141 | Switzerland, Zurich |  | - |  | PDC-8 | OXA-50 | oprD_V359L | - | >= 64 | 64 | 8 | 16 | 32 | 4 | 16 | 16 | 128 | >16 | >16 | 8/4 | > 8/4 | >16 | >4 | 2/4 | 32 | > 16/8 | 64/4 |
| 142 | Switzerland, Zurich | 357 | VIM-5 | VEB-14 | PDC-11 | OXA-486 | OXA-10 | MBL+ESBL | >= 64 | 64 | 32 | 16 | 32 | 16 | 16 | 16 | 128 | >16 | >16 | > 8/4 | > 8/4 | >16 | >4 | > 4/4 | >32 | > 16/8 | > 64/4 |
| 145 | Ethiopia | 712 | - | - | PDC-22 | - | rmtB4, PAU-1/TEM-1 | - | 16 | 2 | 1 | 2 | 8 | 16 | 2 | 16 | 32 | >16 | 8 | 2/4 | 8/4 | 16 | >4 | 2/4 | 16 | 16/8 | 16/4 |

ST, sequence type; ATM, aztreonam; CAZ, ceftazidime; C/T, ceftolozane-tazobactam; C/A, ceftazidime-avibactam; FEP, cefepime; IMP, imipenem; I/R, imipenem-relebactam; P/T, piperacillin-tazobactam
